# Supplementary material for: Functional Gastrointestinal Disorders in Outpatients Aged up to 12 Months: A French Non-Interventional Study
Source: Int J Environ Res Public Health. 2020 Jun 5;17(11):4031. doi: 10.3390/ijerph17114031 (PMC7312775; doi:10.3390/ijerph17114031)
Supplement: Supplementary file 1 [file ijerph-17-04031-s001.pdf]

Supplementary Materials

Table S1. Digestive and other symptoms in infants—Regurgitation.

**Symptoms of Regurgitation**		**Total** *N* **= 1722**
Regurgitation over the past month according to physician's diagnosis	Number	1688
	Missing data	34
	No	931 (55.2%)
	Yes	757 (44.8%)
Vandenplas score	Number	1194
	Missing data	528
	No regurgitation	370 (31.0%)
	≤5 regurgitations/day of small volume	416 (34.8%)
	>5 regurgitations/day of >1 coffee spoon	263 (22.0%)
	>5 regurgitations/day of ±half of the feedings in <half of the feedings	64 (5.4%)
	Continuous regurgitations of small volumes >30 min after each feeding	68 (5.7%)
	Regurgitation of half to complete volume of a feeding in at least half of the feedings	9 (0.8%)
	Regurgitation of the "complete feeding" after each feeding	4 (0.3%)
Mean daily number of regurgitation episodes	Number	943
	Missing data	779
	Mean ± SD	3.7 ± 3.3
	Median	3
	Min–Max	0–30
At least 2 daily regurgitation episodes over 3 weeks	Number	824
	Missing data	898
	No	137 (16.6%)
	Yes	687 (83.4%)
Other symptoms	Number	821
	Missing data	901
	No	662 (80.6%)
	Yes	159 (19.4%)
Suspicion or presence of an organic cause	Number	814
	Missing data	908
	No	759 (93.2%)
	Yes	55 (6.8%)

SD (standard deviation).

Table S2. Digestive and other symptoms in infants—Colic.

**Symptoms of Colic**		**Total** *N* **= 1722**
Colic over the past month according to physician's diagnosis	Number	1706
	Missing data	16
	No	1197 (70.2%)
	Yes	509 (29.8%)
Inconsolable crying, fussing or irritability over the past month, without obvious cause	Number	1577
	Missing data	145
	No	1176 (74.6%)
	Yes	401 (25.4%)
Infant age <5 months at the beginning and end of symptoms	Number	405
	Missing data	1317
	No	37 (9.1%)
	Yes	368 (90.9%)
Mean duration of episodes of unexplained irritability, complaints or crying (hours per day)	Number	425
	Missing data	1297
	Mean ± SD	2.563 (2.026)
	Median	2.00
	Min–Max	0.00–18.00
Number of days a week with unexplained episodes of irritability, complaints or crying	N	419
	Missing data	1303
	Mean ± SD	5.7 (1.7)
	Median	7
	Min–Max	0 - 7
Unexplained episodes of irritability, complaints or crying ≥3 h per day, at least 3 days per week	Number	431
	Missing data	1291
	No	183 (42.5%)
	Yes	248 (57.5%)
Symptoms of growth retardation in infant; fever, or disease	Number	432
	Missing data	1290
	No	421 (97.5%)
	Yes	11 (2.5%)
Suspicion or presence of an organic cause	Number	431
	Missing data	1291
	No	396 (91.9%)
	Yes	35 (8.1%)

SD (standard deviation).

Table S3. Digestive and other symptoms in infants—Functional diarrhea.

Symptoms of Functional Diarrhea		Total *N* = 1722
Diarrhea over the past month according to physician's diagnosis	Number	1689
	Missing data	33
	No	1556 (92.1%)
	Yes	133 (7.9%)
Stools more liquid than usual	Number	1574
	Missing data	148
	No	1419 (90.2%)
	Yes	155 (9.8%)
Daily number of loose or liquid tools	Number	171
	Missing data	1551
	Mean ± SD	3.8 (1.9)
	Median	4
	Min–Max	1–11
Symptoms that last more than 4 weeks	Number	174
	Missing data	1548
	No	96 (55.2%)
	Yes	78 (44.8%)
Onset of symptoms between the 6th and the 60th month of the infant's life	Number	173
	Missing data	1549
	No	27 (15.6%)
	Yes	70 (40.5%)
	Non applicable	76 (43.9%)
Growth retardation in infant	Number	173
	Missing data	1549
	No	164 (94.8%)
	Yes	9 (5.2%)
Suspicion or presence of an organic cause	Number	171
	Missing data	1551
	No	155 (90.6%)
	Yes	16 (9.4%)

SD (standard deviation).

Table S4. Digestive and other symptoms in infants—Functional constipation.

Symptoms of Functional Constipation		Total *N* = 1722
Functional constipation over the past month according to physician's diagnosis	Number	1682
	Missing data	40
	No	1359 (80.8%)
	Yes	323 (19.2%)
Significant decrease in stool frequency and change in stool consistency	Number	1560
	Missing data	162
	No	1257 (80.6%)
	Yes	303 (19.4%)
Number of stools per week	Number	323
	Missing data	1399
	Mean ± SD	4.2 (3.5)
	Median	3
	Min–Max	1–28
Two or fewer defecations per week	Number	325
	Missing data	1397
	No	243 (74.8%)
	Yes	82 (25.2%)
History of excessive stool retention and avoidance over at least one month	Number	326
	Missing data	1396
	No	240 (73.6%)
	Yes	86 (26.4%)
History of painful or hard bowel movements over at least one month	Number	327
	Missing data	1395
	No	130 (39.8%)
	Yes	197 (60.2%)
History of large-diameter stools over at least one month	Number	327
	Missing data	1395
	No	243 (74.3%)
	Yes	84 (25.7%)
Presence of a large fecal mass in the rectum over at least one month	Number	325
	Missing data	1397
	No	283 (87.1%)
	Yes	42 (12.9%)
Suspicion or presence of an organic cause	Number	326
	Missing data	1396
	No	318 (97.5%)
	Yes	8 (2.5%)

SD (standard deviation).
